# Functional Outcomes in Cauda Equina Syndrome Beyond 48 hours Window: A Case Series

**DOI:** 10.1155/cris/1906412

**Published:** 2025-10-14

**Authors:** Roshan Chaudhary, Aashis Poudel, Aashish Giri, Dinuj Shrestha, Rupesh Raut

**Affiliations:** Department of Surgery, Patan Academy of Health Sciences, Lalitpur, Nepal

**Keywords:** Cauda equina syndrome, surgical decompression, treatment outcome

## Abstract

Cauda equina syndrome (CES) results from compression of the cauda equina (CE) nerve roots and presents with a spectrum of neurological deficits. We report four cases of CES with symptom durations ranging from 3 days to 1 month at presentation. The clinical features included urinary incontinence, reduced perianal sensation, lower limb weakness, and erectile dysfunction in select cases. Despite delays in surgical intervention for some patients, all demonstrated significant postoperative improvement in bladder function and lower limb strength. Bladder sensation began to recover within 5–20 days postoperatively, with complete bladder function restoration achieved between 10 and 120 days. While early surgical decompression remains the standard for optimal outcomes, these cases suggest that meaningful recovery is still possible following delayed intervention. Prompt diagnosis and timely surgery, even in late-presenting cases, may improve functional outcomes. Our series reports early postoperative timelines for recovery and aligns observations with key domains from CES core outcome set in low- and middle-income countries.

## 1. Introduction

Cauda equina syndrome (CES) accounts for approximately 2.7 cases per 100,000 individuals annually [1, 2]. The causes of CES are lumbar disc herniation, spinal stenosis, cysts, fractures, or tumors. CES presents with bladder and/or bowel dysfunction, reduced sensation in the saddle area, or sexual dysfunction with possible neurological deficit in the lower limb [1]. CES is diagnosed clinically, which requires imaging support usually by an magnetic resonance imaging (MRI) scan [3]. Surgical decompression should be done within 48 h of the symptoms to prevent serious neurological sequelae [3]. We report four cases of CES with variable symptom durations at presentation, each achieving functional recovery despite delayed surgical intervention.

We present four cases of cauda equina (CE) according to CARE guidelines [4].

### 1.1. Case Series

#### 1.1.1. Case 1

A 31-year-old male presented with a 1-month history of numbness and tingling in his left leg, increased urinary frequency, and incontinence. Despite taking tamsulosin for a month, his symptoms persisted, prompting evaluation at our center. He denied perianal sensory loss and erectile dysfunction.

Neurological examination showed intact sensation and normal muscle tone. Motor strength was 5/5 in the bilateral L2–L4 distributions. In L5, power was 4/5 on the right and 5/5 on the left. Right ankle jerk was diminished. The straight leg raise test (SLRT) was negative bilaterally. Anal sphincter tone was reduced.

**Figure 1:**
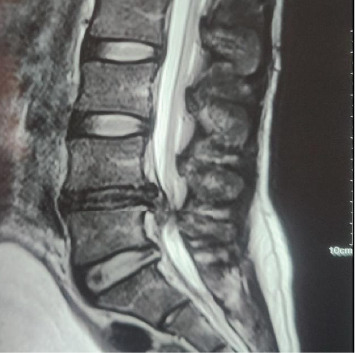
MRI showing broad-based central and right paramedian disc extrusion with caudal migration, causing moderate central canal stenosis at L4-L5 level.

MRI of the lumbosacral spine revealed a broad-based central and right paramedian disc extrusion with caudal migration at the L4–L5 level, resulting in moderate central canal stenosis (Figure 1).

A diagnosis of CES was made, and the patient underwent L4 laminectomy with L4–L5 discectomy. Partial return of bladder sensation occurred by postoperative day 20, although dribbling persisted. With bladder training, intermittent self-catheterization, and physiotherapy over 2 months, bladder function fully recovered. Right lower limb weakness showed gradual improvement but did not fully resolve. The patient was able to return back his day-to-day activities.

#### 1.1.2. Case 2

A 40-year-old male presented with a history of constant dull lower back pain radiating to the left lower limb for 4 days, associated with a burning sensation. He also reported constipation, urinary dribbling, and decreased perianal sensation during the same period. Erectile dysfunction was noted for the past 3 days.

Neurological examination revealed decreased sensation in the left L5–S1 dermatome and reduced perianal sensation (S5) on the left. Motor strength was 5/5 bilaterally across all myotomes except for 4/5 in the left L4 and L5 distributions. Anal tone was normal.

**Figure 2:**
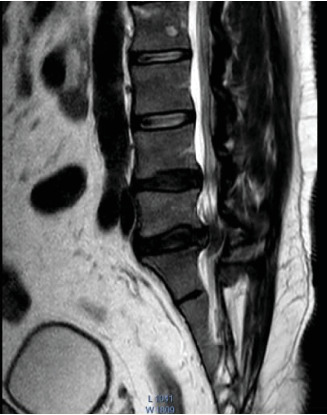
MRI showing posterior bulge at L5-S1 level causing severe spinal canal stenosis with compression of cauda equina nerve root.

MRI of the lumbosacral spine demonstrated a posterior disc bulge at the L5–S1 level causing severe spinal canal stenosis with compression of the CE nerve roots (Figure 2).

The patient underwent decompressive L5 laminectomy and L5–S1 discectomy. Postvoid residual urine volume was 5 mL, and the maximal cystometric capacity was 67 mL on ultrasonography.

Urinary sensation returned within 1 week postoperatively. By 2 weeks, the patient could void without difficulty or leakage and did not require self-catheterization. Physiotherapy and pelvic floor muscle strengthening exercises contributed to the full restoration of urinary function. He returned to work and routine daily activities without restrictions

#### 1.1.3. Case 3

A 46-year-old female presented with urinary retention for 4 days, accompanied by constipation and decreased perianal sensation. She had a 1-year history of lower back pain that had not been evaluated and was managed with analgesics.

Neurological examination revealed sensory loss in the perineal region and posterosuperior thighs. Muscle tone was normal. Motor strength was 5/5 in the L2, L3, L4, and L5 myotomes bilaterally. The right ankle jerk reflex was diminished. SLRT was normal, and anal sphincter tone was preserved.

**Figure 3:**
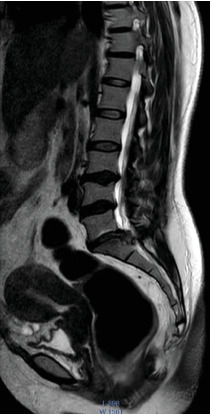
MRI showing diffuse disc bulge at multiple lumbar levels, caudal migration of extruded disc at S1 vertebral level causing compression of adjacent nerve roots with severe spinal canal stenosis.

MRI of the lumbosacral spine showed a diffuse disc bulge at multiple lumbar levels with caudal migration of an extruded disc at the S1 vertebral level, resulting in adjacent nerve root compression and severe spinal canal stenosis(Figure 3).

She underwent L5 laminectomy and L5–S1 discectomy.

Urinary sensation began to return 5 days after surgery, but urinary retention persisted. At discharge, her postvoid residual urine volume was 50 mL. She managed bladder emptying with clean intermittent self-catheterization and underwent pelvic floor muscle strengthening physiotherapy. Complete recovery of bladder function was achieved 4 months after surgery. She resumed independent activities of daily living and returned to her premorbid roles.

#### 1.1.4. Case 4

A 34-year-old male presented with progressive lower back pain of 6 months' duration, radiating to both lower limbs. He also reported a pins-and-needles sensation in the lower limbs and diminished perineal sensation. Additionally, he had experienced urinary dribbling for 3 days and one episode of fecal incontinence.

On examination, the patient was unable to lie supine due to pain. There was no spinal tenderness, and perianal sensation was preserved. Motor examination revealed left-sided L4 myotome strength of 4/5 and right-sided strength of 5/5. L5 myotome strength was 3/5 bilaterally. Sensory assessment showed decreased sensation at the L3, L4, and L5 dermatomes (left: 1/2 and right: 2/2) and preserved sensation at L1 and L2 bilaterally (2/2). Anal sphincter tone was normal.

**Figure 4:**
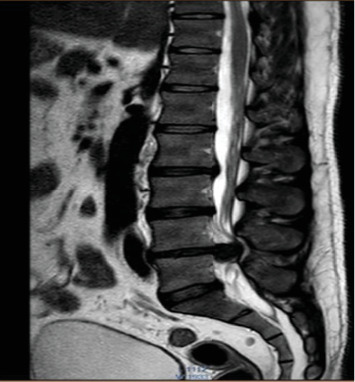
T2-weighted MRI showing posterior disc bulge at L4-L5 level with extrusion leading to severe narrowing of the spinal canal with compression of cauda equina nerve root.

MRI of the lumbosacral spine revealed a posterior disc bulge at the L4–L5 level with extrusion, causing severe spinal canal narrowing and compression of the CE nerve roots (Figure 4).

The patient underwent an L4–L5 discectomy. Intraoperatively, a large sequestrated disc was noted, severely compressing the nerve roots, which appeared swollen and edematous.

Urinary sensation returned 7 days postoperatively. The patient no longer required catheterization and was able to void spontaneously 10 days after surgery. He resumed his activities of daily living and returned to routine activities.

## 2. Discussion

We reported four cases of CES, with symptom durations ranging from 3 days to 4 months prior to diagnosis. All patients were evaluated clinically and radiologically, diagnosed with CES, and underwent surgical decompression. Notably, all patients regained urinary function postoperatively (Table 1).

CES is a neurosurgical emergency that necessitates prompt surgical decompression to avoid permanent neurological damage [1]. It arises from compression of the CE nerve roots, commonly due to lumbar disc herniation, spinal stenosis, trauma, tumors, or inflammatory disorders [1].

Clinically, CES presents with lower limb weakness, areflexia, hypotonia, sensory disturbances, bladder and bowel dysfunction, perineal numbness, and sciatica [5]. It is typically categorized into CES-incomplete (CES-I) and CES-retention (CES-R). CES-I patients retain some bladder sensation, whereas CES-R is marked by complete urinary retention [3].

Early surgical intervention—ideally within 48 h of symptom onset—is associated with improved neurological and bladder function outcomes [6]. Hogan et al. [7] reported that delayed surgery was linked with increased inpatient mortality, a higher complication rate, and nonroutine discharge.

In our case series, outcomes varied with timing of surgery. One patient presented after a month of symptoms and achieved full bladder function recovery 2 months postoperatively. Another presented 4 days after onset of urinary retention and took 4 months to regain bladder control. The fourth case, who underwent decompression within 3 days, achieved full recovery within 10 days. These findings suggest that even beyond the recommended 48-h window, surgical decompression can still result in meaningful neurological improvement. However, delayed interventions may lead to prolonged recovery periods.

Several mechanisms may explain functional recovery despite delayed decompression. Factors such as the degree of nerve root compression, extent of ischemic injury, and preoperative residual function influence outcomes. If ischemic insult is mild, or compression is partial, nerve roots may retain the potential for recovery. Additionally, neuroplastic changes, combined with early physiotherapy and bladder retraining, may support functional restoration [8].

Nonetheless, delays in treatment increase the risk of permanent deficits and significantly affect the quality of life. Long-term complications include urinary and fecal incontinence, sexual dysfunction, and persistent sciatica [9]. Around 20% of CES patients require prolonged support due to catheterization, colostomy, rehabilitation, and psychological issues such as depression and unemployment. Therefore, comprehensive preoperative counseling is critical to inform patients about the potential for enduring impairments and to set realistic postoperative expectations [7]. In relation to large prospective work from high-income settings demonstrating beneficial outcomes despite delayed presentation, our case series complements that literature by providing granular early timelines for return of bladder sensation and continence after late decompression, by aligning observations with CES core outcome set domains, and by outlining a pragmatic rehabilitation approach.

## 3. Conclusion

We reported four cases of CES with symptom durations ranging from 3 days to 4 months, highlighting the variability in presentation and clinical outcomes. Diagnosis was established based on clinical features and MRI findings, and all patients underwent surgical decompression. Although surgery was performed beyond the recommended 48 h window in all cases, significant improvement in bladder function was observed. Three patients experienced early complete resolution, while one patient showed delayed recovery. Supportive measures such as bladder training, self-intermittent catheterization, and pelvic floor muscle strengthening contributed substantially to functional restoration. However, one patient continued to experience persistent motor deficits. Our case series suggests that meaningful recovery is still possible with surgical intervention beyond the 48 h threshold, challenging the rigid application of current timing guidelines for CES management in low- and middle-income country setting.

## Figures and Tables

**Table 1:** Clinical profile and recovery outcomes of patients with cauda equina syndrome undergoing lumbar decompression surgery.

Case	Presented to our center (days)	Surgery	Return of urinary sensation (days)	Complete bladder function (days)
1	30	L4 laminectomy and L4-L5 discectomy	20	60
2	4	L5 lumbar laminectomy and L5–S1 discectomy	7	14
3	4	L5 laminectomy and L5–S1 discectomy	5	120
4	3	L4-L5 discectomy	7	10

## Data Availability

Data sharing is not applicable to this article as no new datasets were generated or analyzed during the current study.
